# Targeting HO-1 by Epigallocatechin-3-Gallate Reduces Contrast-Induced Renal Injury via Anti-Oxidative Stress and Anti-Inflammation Pathways

**DOI:** 10.1371/journal.pone.0149032

**Published:** 2016-02-11

**Authors:** Zhao Gao, Yu Han, Yunhui Hu, Xiaoyan Wu, Yongbin Wang, Xiaoqun Zhang, Jinjuan Fu, Xue Zou, Jun Zhang, Xiongwen Chen, Pedro A. Jose, Xi Lu, Chunyu Zeng

**Affiliations:** 1 Department of Cardiology, Daping Hospital, The Third Military Medical University, Chongqing, P.R. China; 2 Chongqing Institute of Cardiology, Chongqing Key Laboratory for Hypertension Research, Chongqing, P.R. China; 3 Department of Medicine, Division of Renal Disease and Hypertension, The George Washington University School of Medicine & Health Sciences, Washington, DC, United States of America; 4 Department of Physiology, The George Washington University School of Medicine & Health Sciences, Washington, DC, United States of America; Instituto Nacional de Cardiologia I. Ch., MEXICO

## Abstract

Both oxidative stress and inflammation are involved in the pathogenesis of contrast-induced nephropathy (CIN). Epigallocatechin-3-gallate (EGCG), a purified catechin from green tea, has antioxidant and anti-inflammatory effects. However, it is unknown whether or not EGCG is effective in treating CIN. Our present study found that intravenous administration of EGCG, either before or just after the establishment of CIN, had a protective effect, determined by normalization of serum creatinine and blood urea nitrogen levels, improvement in renal histopathological scoring and alleviation of apoptosis, accompanied by decreased oxidative stress and inflammation. Because EGCG is a potent inducer of the antioxidant heme oxygenase-1 (HO-1), we studied HO-1 signaling in CIN. HO-1 levels were increased in CIN; treatment with EGCG further increased HO-1 levels, accompanied by an increase in Nrf2, a regulator of antioxidant proteins. Interestingly, blockade of HO-1 with protoporphyrin IX zinc(II) (ZnPP) prevented the protective effect of EGCG on CIN. ZnPP also blocked the ability of EGCG to increase the activity of an antioxidant (superoxide dismutase), and decrease markers of oxidative stress (myeloperoxidase and malondialdehyde) and inflammation (myeloperoxidase and IL-1β), indicating that HO-1 is the upstream molecule that regulates the EGCG-mediated protection. To determine further the role of HO-1 on the EGCG-mediated inhibition of inflammation, we studied the effect of EGCG on the NLRP3 inflammasome, an upstream signaling of IL-1β. EGCG down-regulated NLRP3 expression, which was blocked by ZnPP, indicating that HO-1 links EGCG with NLRP3. Therefore, EGCG, via up-regulation of HO-1, protects against CIN by amelioration of oxidative stress and inflammation.

## Introduction

Contrast-induced nephropathy (CIN) continues to be a common iatrogenic cause of acute kidney injury (AKI) after exposure to iodinated contrast medium (CM), e.g., during percutaneous coronary intervention (PCI), despite the tailored preventive strategies that include risk stratification for the individual patient, hydration, newer and safer CM and additional preventive methods (e.g., N-acetylcysteine [1], sodium bicarbonate [2], fenoldopam [3], statins [4,5], limb ischemic preconditioning [6], and preemptive hemodialysis [7]). Patients susceptible to CIN have comorbidities such as diabetes, chronic kidney disease, heart failure, and advanced age. The incidence of CIN in these patients can reach 20–30% [8,9], increasing the potential for the development of long-term loss of renal function [9,10]. To date, there is still lack of evidence-proved prevention or treatment of CIN.

Although the precise mechanisms that cause CIN are not fully understood, there seems to be a consensus that acute ischemia/hypoxia caused by CM or its toxicity per se, leads to acute tubular necrosis. Oxidative stress and inflammation have been implicated in the pathogenesis of CIN [8,9]. CM filtered by the glomerulus, actively taken up by renal tubular cells and retained within the cells and peritubular space, especially in patients with chronic kidney impairment, not only has a direct toxic action on tubular cells, increasing oxygen consumption, but also induces vasoconstriction of the vasa recta, decreasing oxygen delivery, inducing a state of hypoxia. CM triggers a series of reactions that lead to the release of free radicals, causing cellular damage and initiating the vicious cycle of oxidative stress and inflammation. Thus, a possible treatment strategy could involve the use of medications that target the regulators of both renal oxydative stress and inflammation.

Epigallocatechin-3-gallate (EGCG), a purified and active component of green tea, has been reported to possess both antioxidant and anti-inflammatory properties in the treatment of diseases in the cardiovascular system [11], lung [12], liver [13,14] and kidney [15–23]. The protective effects of EGCG have been reported in various acute and chronic kidney diseases, including obstructive nephropathy [15], cisplatin nephrotoxicity [16,17], renal ischemia-reperfusion injury [18], kidney damage induced by extracorporeal circulation [19], diabetic nephropathy [20,21], lupus nephritis [22], and chronic glomerulonephritis [23]. We hypothesized that EGCG may exert a protective effect on CIN. We found that the beneficial effect of EGCG in a CIN rat model involved an EGCG-mediated up-regulation of HO-1 that mitigated both oxidative stress and inflammation.

## Materials and Methods

### Animal care

Male Sprague-Dawley (SD) rats, weighing 220–250g, were used for the experiments. The SD rats had free access to tap water and standard rat chow on a 12-h-light/dark cycle. The experimental protocol was approved by the Animal Care and Use Committee of Third Military Medical University (Permit Number: 2013–12). All surgery was performed under sodium pentobarbital anesthesia and all efforts were made to minimize suffering.

### Reagents

Indomethacin, Nω-nitro-L-arginine methyl ester hydrochloride (L-NAME), and EGCG were bought from Aladdin (Shanghai, China), iopromide from Bayer HealthCare China (Beijing, China), protoporphyrin IX zinc(II) (ZnPP) from Sigma (St. Louis, MO), and tin protoporphyrin IX dichloride (SnPP) from Tocris Bioscience (Bristol, UK). Rabbit anti-HO-1 polyclonal antibody, rabbit anti-nuclear factor E2-related factor 2 (Nrf2) polyclonal antibody, rabbit anti-histone H3 polyclonal antibody, and rabbit anti-nucleotide-binding oligomerization domain receptor (NOD-like receptor, NLR) subset 3 (NLRP3) polyclonal antibody were purchased from Proteintech Group Inc. (Chicago, IL), rabbit anti-GAPDH from Goodhere Technology (Hangzhou, China), and goat anti-rabbit IRDye 800 CW from LI-COR Biosciences (Lincoln, NE). Sodium citrate-EDTA antigen retrieval solution, immunostaining blocking solution and Cy3-labeled goat anti-rabbit IgG were purchased from Beyotime (Shanghai, China).

### Establishment of rat CIN model

The rat CIN model was established as previously reported, with minor modifications [24,25]. The rats were deprived of water 24h before the acute insult. After the rats were anesthetized with an intraperitoneal injection of sodium pentobarbital (50mg/kg body wt), and placed on a heating table to maintain body temperature at 37°C, the left external jugular vein was cannulated with PE-10 tubing. In the CM group, indomethacin in ethanol (10mg/kg body wt), L-NAME in normal saline (10mg/kg body wt), and iopromide (1.8g(I)/kg body wt), were sequentially injected at 15min intervals. The vehicle group received the same amount of solvents.

EGCG (5, 10, 20mg/kg body wt) in normal saline was given intravenously at the indicated time-points. ZnPP, a HO-1 inhibitor, in normal saline (30mg/kg body wt), was given intraperitoneally 7h before EGCG and other treatments[26,27]. Then, the rats were allowed to recover at the indicated times (24–72h) and continued to have free access to water and rat chow. The rats were kept in metabolic cages for 24h urinary collections.

At the end of experiments, the rats were re-anesthetized with sodium pentobarbital, 100mg/kg body wt. After laparotomy, all available blood was withdrawn from the abdominal aorta. Then, the kidneys were removed and rinsed twice in ice-cold phosphate-buffered saline (PBS). One longitudinal half of the left kidney was fixed in 4% (w/v) paraformaldehyde in PBS for histological assessment. The remaining half was stored at -80°C until use.

### Biochemical assays

Blood sera were separated by centrifugation at 1,200g for 10min and stored at -80°C until use. Serum creatinine (Cr), blood urea nitrogen (BUN), and urinary Cr were measured by an automatic biochemistry analyzer, Analyzer Medical Systems (SaBa-18, Rome, Italy), using commercial kits (ZhongSheng BeiKong Bio-Technology and Science Inc., Beijing, China). The creatinine clearance (CrCl) was calculated according to the formula: CrCl = UV/P: U represents the urinary Cr concentration (μmol/L); V is the total urine volume collected for 24hrs (ml/min); and P is serum Cr concentration (μmol/L) [28].

### Renal histopathological assessment

The longitudinal half of the kidney, fixed in 4% paraformaldehyde for 48h, was subjected to routine dehydration and paraffin embedding. Sections (4μm thick) were deparaffinized and stained with hematoxylin and eosin (H&E). The grading criteria for histopathological scoring of renal medullary damage including tubular vacuolar degeneration/necrosis, tubular casts, and congestion, followed the published methodology with minor modification [25]. Grading of tubular vacuolar degeneration/necrosis under ×400 magnification (scoring 0 to 4) was: no damage (0); number of patchy isolated damage ≤3 (1); damaged area of the microscopic field <10% (2); 10% ~25% (3); and >25% (4). Grading of protein casts under ×200 magnification was: no casts (0); number of casts ≤ 5 (1); casts area <25% of microscopic field (2); between 25% ~50% (3); and >50% (4). The degree of interstitial congestion was graded as: no congestion (0); presence of extravascular erythrocytes under ×400 magnification (1); ×200 magnification (2); ×100 magnification (3); and ×40 magnification (4). The scoring was performed in 10 fields per section from three different sections with the examiner blinded from the experimental protocol. Data were expressed as the average score per field.

### Terminal deoxynucleotidyl transferase dUTP nick end labeling (TUNEL)

Apoptosis in paraffin-embedded kidney sections was detected using an In Situ Cell Death Detection kit (POD; Roche Applied Bio Sciences, Basel, Switzerland), as previously described [29]. Nuclei were identified with DAPI staining. Images under ×200 magnification field were obtained using a fluorescence microscope (Eclipse Ti-U, Nikon Corporation, Tokyo, Japan) at an excitation wavelength of 405nm for DAPI and 488nm for TUNEL. The number of TUNEL-positive nuclei was quantified in 10 fields in renal medulla per section from three different sections with the examiner blinded from the experimental protocol. Data were expressed as the average number of TUNEL-positive nuclei per ×200 magnification field.

### Assays of oxidative stress and inflammatory markers

Renal tissues were homogenized in ice-cold sucrose buffer (pH 7.4) according to the instructions of the assay kit. A marker of lipid peroxidation, malondialdehyde (MDA), and a marker of oxidative stress and inflammation, myeloperoxidase (MPO) were analyzed according to the protocols of commercial assay kits from Jiancheng Bioengineering Institute (Nanjing, China). The activity of the antioxidant, superoxide dismutase (SOD) was measured using a SOD assay kit from Dojindo Laboratories (Kumamoto, Japan). The level of the pro-inflammatory cytokine IL-1β was measured using an IL-1β enzyme-linked immunosorbent assay kit from R&D Systems (Minneapolis, MN). The values were normalized by tissue protein concentration.

### Immunoblotting

Renal tissues were homogenized in lysis buffer containing 20mM Tris-HCl, pH 7.4, 2mM EDTA, pH 8.0, 2mM EGTA, 100mM NaCl, 10μg/ml leupeptin, 10μg/ml aprotinin, 2mM phenylmethylsulfonyl fluoride (PMSF), 1% NP-40, and 2mg/ml aprotinin, and ultrasonicated for 15s, 5 times, on ice. Then, the homogenates were centrifuged at 15,000g for 40min at 4°C. Nuclear and cytosolic fractions were obtained using an extraction kit from Beyotime (Shanghai, China) [30]. Protein concentrations of the samples, measured by Bradford assay, were adjusted to the same final concentration using the lysis buffer. Protein samples were boiled at 100°C for 10min in SDS-containing sample loading buffer and stored at -20°C until use.

Equal amounts of protein were loaded, separated on SDS-PAGE, and transferred onto nitrocellulose membranes. After blocking with 5% (w/v) non-fat milk in TBST (Tris-buffered saline with 0.05% Tween 20) for 2h at room temperature, the membranes were incubated with primary antibody in the appropriate dilutions at 4°C overnight [anti-HO-1 antibody (1:200), anti-Nrf2 antibody (1:200), anti-histone H3 antibody (1:1500), anti-NLRP3 antibody(1:200), and anti-GAPDH antibody (1:500)]. Thereafter, the membranes were washed 3 times with TBST and incubated with secondary antibody, goat anti-rabbit IRDye 800 (1:15000), for 1h at room temperature. The protein bands were visualized using the Odyssey Infrared Imaging System (Li-Cor Bioscience, Bad Homburg, Germany), and quantified using the Quantity One software. Densitometric intensity corresponding to each band was normalized against either cytosolic or nuclear internal reference, GAPDH and histone H3, respectively [31].

### Immunofluorescence microscopy

The kidney sections were deparaffinized and rehydrated. Antigen retrieval was performed by microwave heating in sodium citrate-EDTA antigen retrieval solution. After natural cooling and PBS rinses, the tissue sections were mixed with immunostaining blocking solution for 1h at room temperature to prevent nonspecific antibody binding. Then, the sections were incubated with anti-NLRP3 antibody (1:25) or anti-HO-1 antibody (1:50) at 4°C overnight. After washing with PBS for 5 min 3 times, the sections were incubated with secondary antibody, Cy3-labeled goat anti-rabbit IgG (1:200), at room temperature for 1h. Finally, after washing with PBS (5 min, 3 times), the sections were stained with DAPI before being imaged under a fluorescence microscope (Eclipse Ti-U, Nikon Corporation, Tokyo, Japan) at an excitation wavelength of 405nm for nuclei and 543nm for NLRP3 and HO-1.

### Statistical analysis

All data were analyzed by SPSS 13.0 (Chicago, IL) and presented as mean ± SEM. Data were compared by one-way ANOVA with Bonferroni post-hoc test for multiple comparisons, after checking for normality (Kolmogorov-Smirnov) and homogeneity (Levene). The H&E scores among groups were compared by the nonparametric Kruskal-Wallis test. P<0.05 was considered statistically significant.

## Results

### The renal protective effect of EGCG on CIN

**Fig 1:**
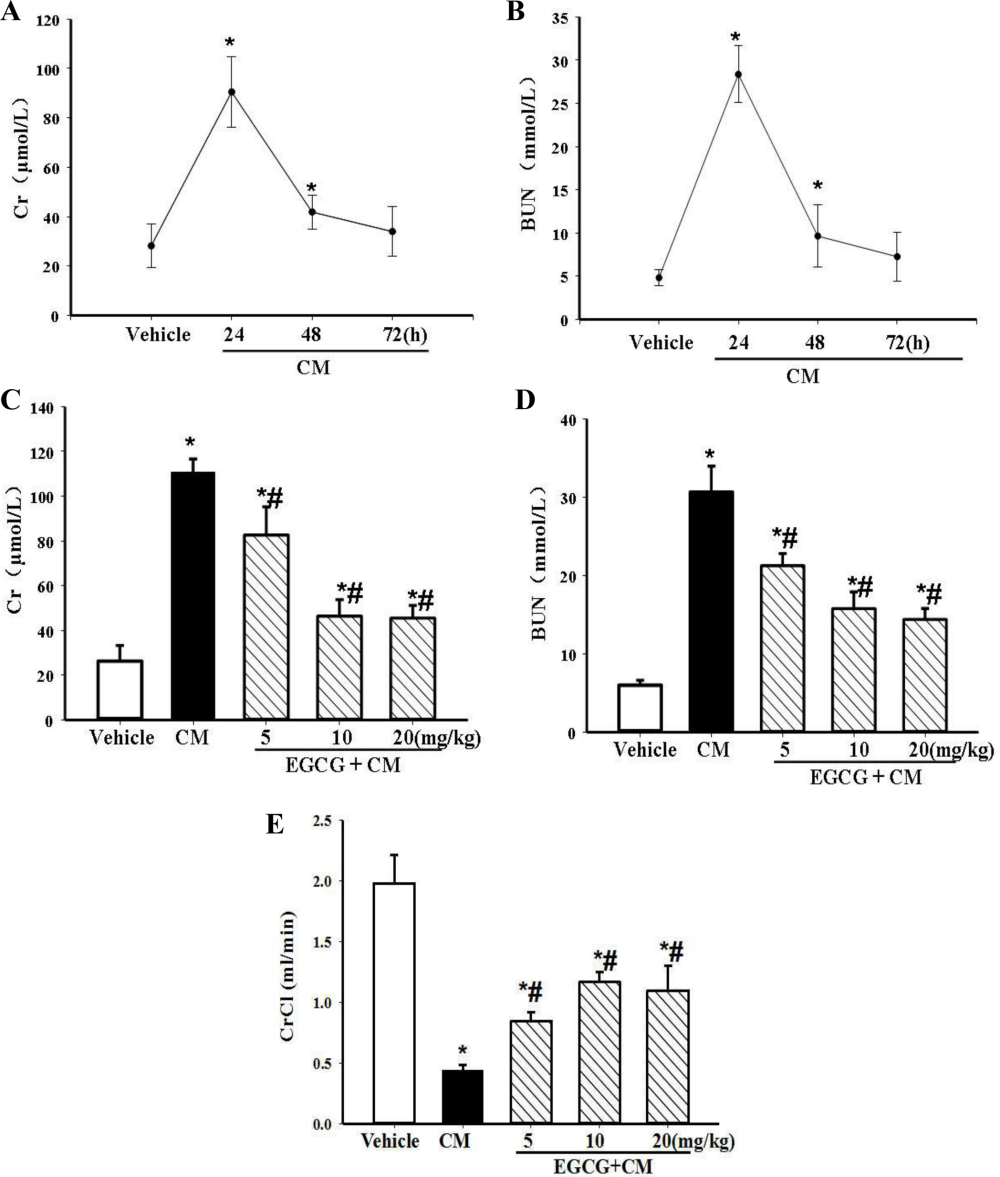
Establishment of CIN in rats and renal protective effect of EGCG on CIN. The rats sequentially received an intravenous infusion of indomethacin, L-NAME, and iopromide to establish CIN. Serum Cr (A) and BUN (B) concentrations were measured 24h, 48h, and 72h after the intravenous injections. Varying dosages of EGCG (5, 10, 20mg/kg body wt) were administered intravenously 15min before the establishment of CIN. Serum Cr (C), BUN (D) and CrCL (E) were measured 24h after the establishment of CIN. (n = 5, * P<0.05 vs. vehicle; # P<0.05 vs. CM).

In the present model, the levels of serum Cr and BUN were found to peak at 24h, decreasing at 48h, and almost receding back to normal at 72h. Thus, the time-point at 24h was used to evaluate renal function in this contrast-induced AKI model (Fig 1A and 1B). Intravenous pretreatment with 5 to 20mg/kg body wt of EGCG was able to reduce the extent of CIN, as assessed by renal function markers, i.e., serum Cr, BUN, and CrCl at 24h after the injury (Fig 1C–1E). The maximal effect of EGCG was found at the dosage of 10mg/kg body wt, which was chosen for subsequent experiments.

**Fig 2:**
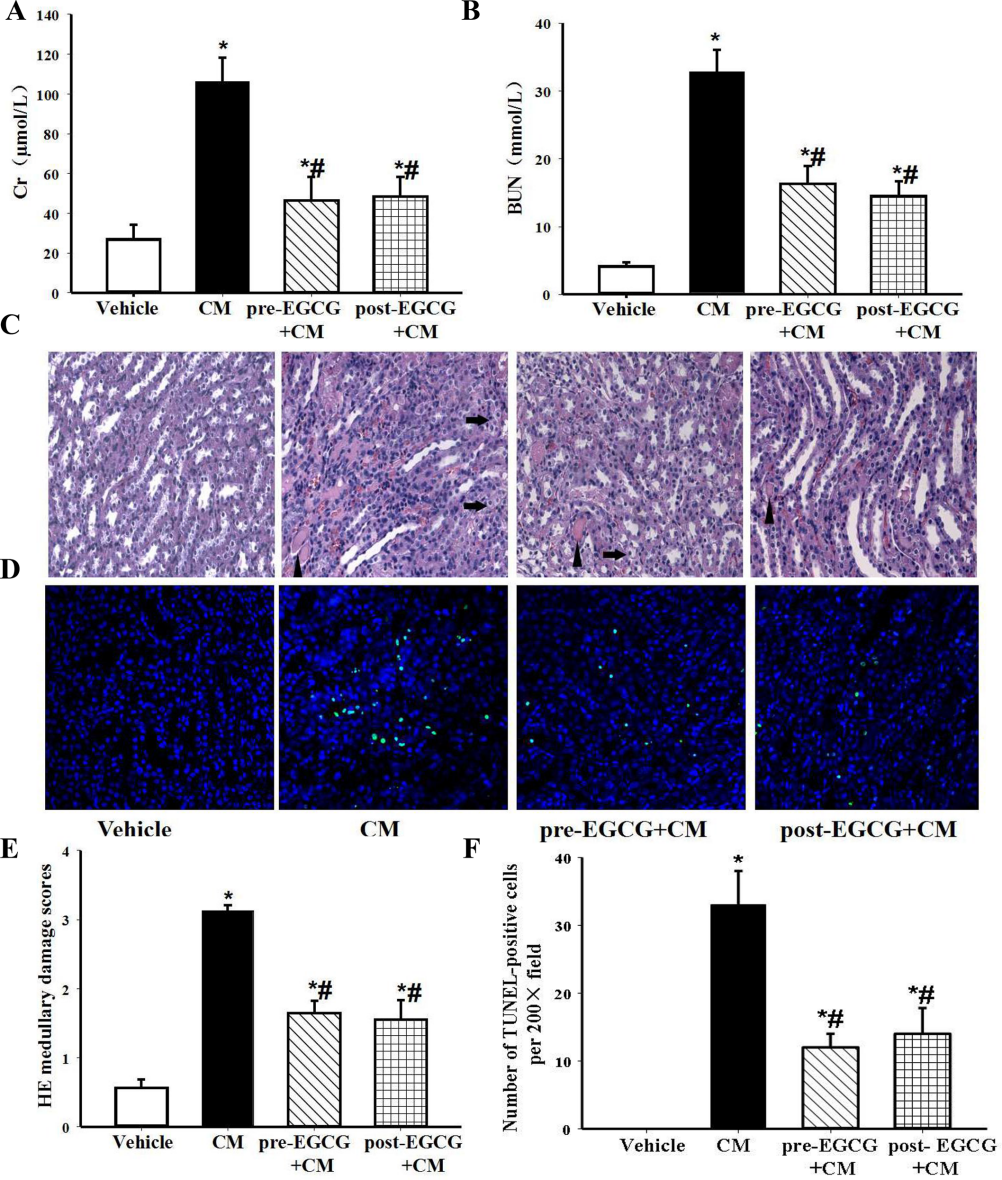
Renal protective effect of EGCG on medullary damage and apoptosis in CIN kidney. EGCG (10mg/kg body wt) was given intravenously 15min before (pre-EGCG+CM) or after the establishment of CIN (post-EGCG+CM). Serum Cr (A) and BUN (B) were measured 24h after the establishment of CIN. (C) Representative images of H&E staining under ×400 magnification in the outer medulla. Arrows showed examples of protein casts and tubular vacuolar degeneration/necrosis. Erythrocytes and infiltration of polymorphonuclear cells could be easily observed in the interstitium. (D) Representative images of TUNEL assay under ×400 magnification in the outer medulla. (E) The histopathologic scores of medullary damage. (F) Quantification of TUNEL-positive nuclei per ×200 field. (n = 5, * P<0.05 vs. vehicle; # P<0.05 vs. CM).

We also determined if the time-window of intervention, before or just after the contrast exposure, made any difference in the severity of CIN. We found that regardless of the time of pre- or post-EGCG treatment, a dosage of 10mg/kg body wt, significantly ameliorated the CIN-associated increase in serum Cr and BUN, as compared with vehicle treatment. There were no differences between these two intervention time-points (Fig 2A and 2B), suggesting that EGCG treatment could be effective in both pre- and post-injection of CM. The protective effects of EGCG on medullary damage and apoptosis were further confirmed by histological examination. In the CM group, CIN induced tubular vacuolar degeneration/necrosis, protein (hyaline) and cellular casts that were associated with erythrocytes and infiltration of polymorphonuclear cells in the interstitium (Fig 2C). The major damages were in the renal outer medulla (location of medullary thick ascending limb, mTAL), rather than in the inner medulla, cortex or conjuction of cortex and medulla (location of the pars recta segment (S3) of the proximal tubule) (S1 Fig), consistent with the reports of Agmon Y [24] and Bird JE [32]. The above-mentioned abnormal changes were significantly restored after treatment with EGCG (10mg/kg body wt). The histopathologic scores of medullary damage were lower in the EGCG-treated groups than the untreated CM group (Fig 2C and 2E). The number of TUNEL-positive nuclei, reflecting the apoptosis, was substantially increased after contrast-injudced AKI, and reduced by EGCG either pre- or post-treatment (Fig 2D and 2F).

### Role of HO-1 in the antioxidant and renal protective effect of EGCG on CIN

**Fig 3:**
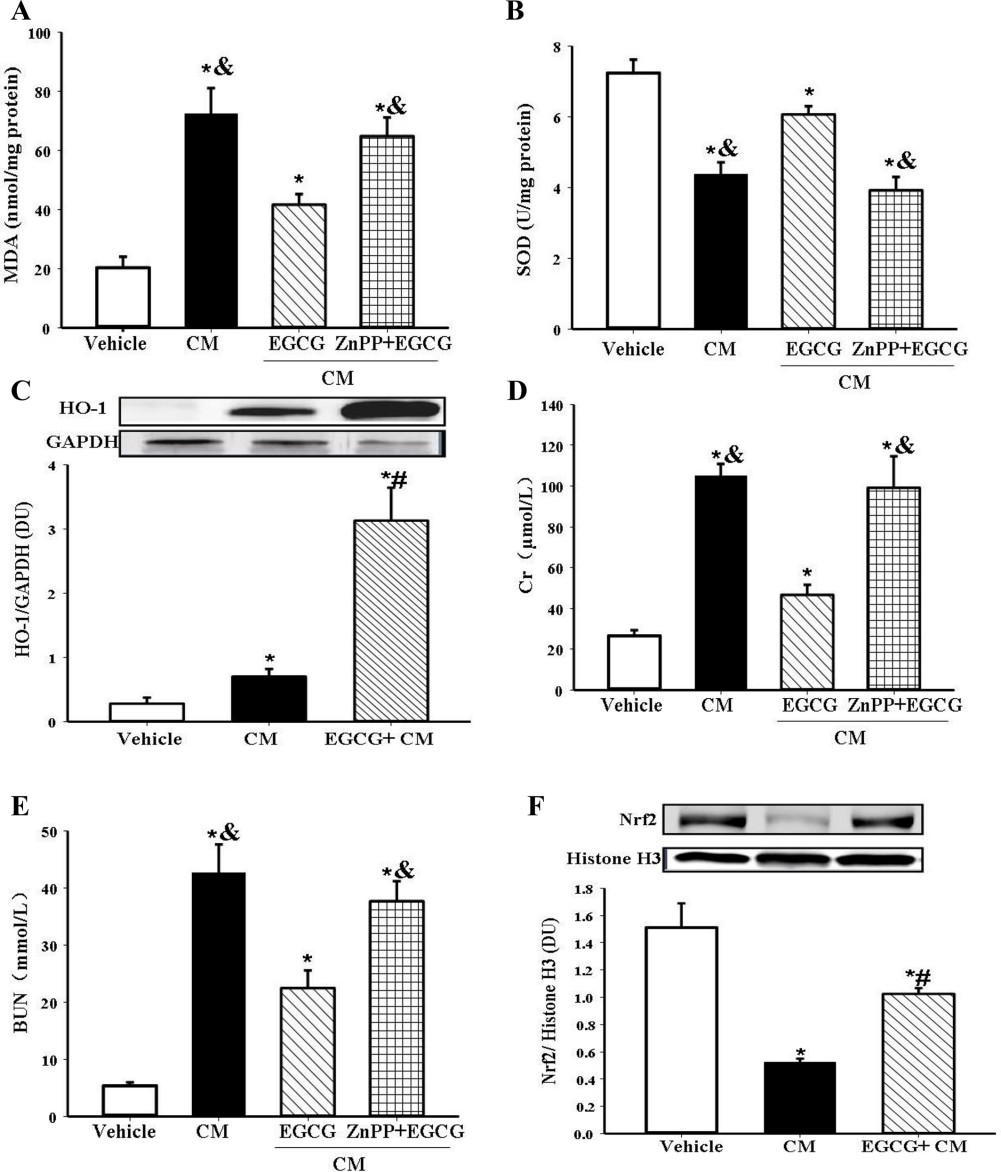
Role of HO-1 in the antioxidant and renal protective effect of EGCG in CIN. EGCG (10mg/kg body wt) was intravenously infused 15min before the establishment of CIN. The HO-1 inhibitor ZnPP (30mg/kg body wt) was injected intraperitoneally 7h before EGCG pretreatment. The rats were sacrificed at 24h after the establishment of CIN. Renal MDA level (A) and SOD activity (B) were measured. Renal HO-1 protein detected by immunoblotting was expressed as the ratio of HO-1 and GAPDH (C). Serum Cr (D), and BUN (E) were determined to evalute the renal function. Nrf2 expression in the nuclear fraction of renal tissue detected by immunoblotting was expressed as the ratio of Nrf2 and histone H3 (F). (n = 5, * P<0.05 vs. vehicle; # P<0.05 vs. CM; & P<0.05 vs. EGCG).

Oxidative stress is an initiator and major component in pathogenesis of CIN [8,9]. To evaluate the effect of EGCG on renal oxidative stress, we measured the renal expression of the oxidative stress marker MDA. CIN was associated with oxidative stress because renal MDA content was increased; the increase was almost normalized by EGCG pretreatment (Fig 3A).

Though EGCG has no effect on baseline reactive oxygen species [15,16], it has been reported to increase the levels of several antioxidant enzymes, including SOD, catalase, glutathione peroxidase (GPx), and heme oxygenase-1 (HO-1) in pathological condictions [17,18,33,34]. We wondered which one is the key enzyme involved in the antioxidant effect of EGCG. We found that the CIN-induced decrease in the activity of SOD was almost normalized by EGCG pretreatment (Fig 3B). However, HO-1, as a stress inducible antioxidant enzyme, has been reported as a powerful cytoprotective protein in several disease states, including that from renal injury [17,18,31]. Therefore, we measured HO-1 levels and found that HO-1 was increased in CIN compared with the vehicle group. While EGCG was reported to have no effect on baseline HO-1 in kidney [35], we found that EGCG treatment increased HO-1 expression to an even higher level in CIN (Fig 3C) both in the cortex and medulla (S2 Fig).

To confirm the key role of HO-1 in the protective effect of EGCG, we studied the effect of EGCG on CIN when HO-1 was inhibited by ZnPP (30mg/kg body wt). ZnPP acts as a competitive inhibitor of HO-1 [26,27]. Blocking HO-1 activity by ZnPP almost completely abrogated the renal protective effect of EGCG on CIN; it reversed the beneficial effect of EGCG on serum Cr, BUN (Fig 3D and 3E), and kidney MDA, SOD (Fig 3A and 3B), indicating that these renal protective and antioxidant effects of EGCG may via HO-1. To reconfirm the role of HO-1 in EGCG’s protective action, another inhibitor SnPP was used to block the activity of HO-1 [36,37]. SnPP (10mg/kg body wt i.p. 2h before EGCG pretreatment) also significantly offsetted the reno-protective effect of EGCG, assessed by serum Cr and BUN (S3 Fig). Although the exact mechanism by which EGCG upregulates HO-1 expression is not clear, Nrf2, a transcription factor of antioxidant genes with antioxidant response element (ARE), has been found as the regulator of HO-1. Nrf2/HO-1 signaling has also been implicated in the protective effect of EGCG on renal injury [17,18]. Indeed, we found that EGCG increased the protein expression of Nrf2 in CIN (Fig 3F), indicating EGCG may increase the expression of HO-1 at the level of transcription.

### Role of HO-1 in the anti-inflammatory effect of EGCG on CIN

**Fig 4:**
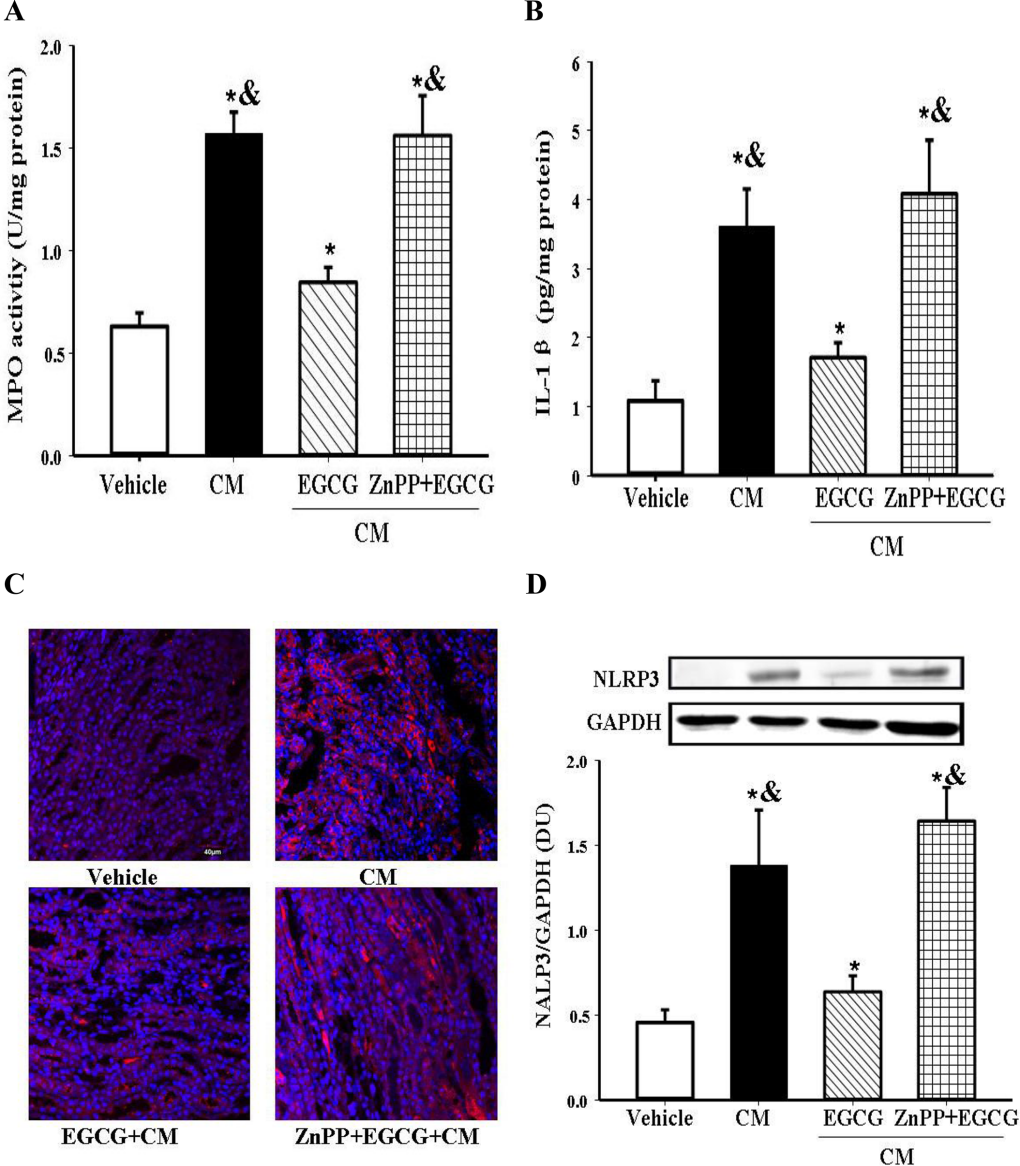
Role of HO-1 in the anti-inflammatory effect of EGCG in CIN. EGCG (10mg/kg body wt) was intravenously infused 15min before the establishment of CIN. The HO-1 inhibitor ZnPP (30mg/kg body wt) was injected intraperitoneally 7h before EGCG pretreatment. The rats were sacrificed at 24h after the establishment of CIN. Renal MPO activity (A) and IL-1βlevel (B) were measured. Renal NLRP3 protein expression was detected by immunofluorescence microscopy (red fluorescence, ×400) (C), and semi-quantified by immunoblotting, expressed as the ratio of NLRP3 and GAPDH (D). (n = 5, * P<0.05 vs. vehicle; # P<0.05 vs. CM; & P<0.05 vs. EGCG).

In addition to oxidative stress, inflammation also plays an important role in the CM-induced renal damage [8,9]. Our present study found that signs of inflammation in CIN were increased, as indicated by increased MPO (marker of both oxidative stress and inflammation) and IL-1β (marker of inflammation) levels (Fig 4A and 4B). EGCG treatment reduced the increased MPO and IL-1β levels in CIN (Fig 4A and 4B). To determine the effect of EGCG on inflammatory signaling, we measured the expression of NLRP3 inflammasome, which is upstream of IL-1β. We found that NLRP3 expression that was increased in CIN was reduced by EGCG treatment (Fig 4C and 4D), indicating that EGCG, via NLRP3, regulated the IL-1β levels.

As indicated in the above-mentioned results, amelioration of oxidative stress and inflammation is involved in the protective effect of EGCG. Although the lower level of inflammation induced by EGCG may be subsequent to its antioxidant action, we wondered if there is a molecular connection between actions of EGCG on reactive oxygen species production and inflammation. To test the hypothesis that HO-1 plays a pivotal role in the EGCG-mediated negative regulation of inflammation, we studied the effect of ZnPP, a HO-1 inhibitor. We found that ZnPP prevented the ability of EGCG to reduce the increased activity and content of the inflammatory markers, MPO and IL-1β in CIN (Fig 4A and 4B). Moreover, the ability of ZnPP to decrease NLRP3 protein expression was evident by immunofluorescence microscopy and immunoblotting (Fig 4C and 4D). Taken together, these results indicate that HO-1 is the key target of EGCG in reducing both the oxidative stress and inflammation in CIN.

## Discussion

CIN is the third leading cause of AKI, accounting for 10–13% of cases in hospitalized patients [38]. The renal tubular damage in CIN is caused by a decrease in renal blood flow and direct cytotoxic effect. It is hard to establish a contrast-induced AKI in a normal rat without predispositions. Homeostasis of medullary oxygenation may depend on prostanoids and nitric oxide. Impaired endothelium-derived vasorelaxation in diabetes mellitus, hypertension, atherosclerosis and heart failure, results in regional hypoxia. Indomethacin, a cyclooxygenase inhibitor, and L-NAME, a nitric oxide synthase inhibitor in this model were used as predispositions to decrease the production of prostanoids and nitric oxide respectively for later contrast-induced AKI, which was thoroughly studied by Agmon Y [24]. This method has been widely used in experimental studies of CIN [26,30,39]. As low-osmotic CM, rather than a high osmotic CM, is more commonly used in clinic, therefore, iopromide, a low osmotic CM, was chosen to establish the model of CIN.

Previous studies have shown a beneficial effect of green tea on dextran sulfate sodium- or cisplatin-induced nephropathy [40,41]. However, there are no reports about the effect of EGCG in CIN. In the present study, we demonstrated that the intravenous administration of EGCG, either before or just after the establishment of CIN, had a protective effect, assessed by measurements of serum Cr and BUN, H&E histopathological scoring and apoptosis.

Oxidative stress and inflammation are two major factors involved in the pathogenesis of CIN. Oxidative stress can initiate the tubular injury and induce inflammation that in turn causes oxidative stress, resulting in a vicious cycle, augmenting the sterile damage. Sterile inflammation is a reaction of the immune system in response to tissue injury that is essential for clearance of cell debris and tissue repair. However, uncontrolled excessive and/or prolonged activation of inflammation causes tissue damage, and contributes to the pathogenesis of AKI that eventually leads into chronic kidney disease [42–44]. In our study, EGCG significantly reduced both oxidative stress and inflammation in the kidney, attested by the reduction in MDA level, increase in SOD activity, and reduction in MPO activity and IL-1β level.

Interstitial congestion and hemoglobin oxidation are typical of acute tubular necrosis caused by CM. Heme (iron(II)-protoporphyrin IX), released from hemoglobin following hemolysis, possesses pro-inflammatory and pro-oxidative properties. It is a hallmark of extensive tissue damage, playing a central role in the pathogenesis of malaria, sepsis, sickle cell disease [33,45,46], and AKI in the elderly [47]. Endogenous molecules from damaged cells are essential in the auto-inflammatory response [33,43,44]. Innate immune receptors, which are pattern recognition receptors (PRRs), including the widely distributed transmembrane Toll-like receptors (TLRs), provide a sensing network for endogenous ligands [48], like heme and its derivatives [33,45,49–52]. Thus, heme may be an important risk factor in escalating the oxidation and inflammation of CIN. Interestingly, catabolism of heme by HO converts the cytotoxic heme into cytoprotective catabolites, including iron, biliverdin, and carbon monoxide, that have antioxidant and anti-inflammatory properties. HO-1, an inducible isozyme, is activated by heme, oxidants, cytokines, glycated albumin, and other stressors, and may be part of the protective response in many diseases [12,32], including acute lung injury [53], lipopolysaccharide-induced acute liver failure [54], neurodegenerative disorders [55], and AKI [16–18]. In the present study, we found that the protective effect of EGCG on CIN is mediated by HO-1, because blockade of HO-1 activity abolished the protective effect of EGCG.

Although inhibition of PI3k/Akt and MAPK signaling pathways by EGCG has been reported involved in the anti-inflammatory mechanism in ameliorating crescentic glomerulonephritis [56], the NLRs, which are cystolic PRRs, and NLR-activated inflammasome, typically NLRP3, aroused our interest. NLRP3 inflammasone is a central component of innate immunity and the sterile inflammatory response that acts as a guardian, linking damage sensing to the initiation and amplification of the inflammatory response [48,57]. When triggered by bacterial toxins, and environmental or intracellular danger signals, NLRP3 oligomerizes and recruits ASC (apoptosis speck-like protein containing a caspase-recruitment domain). ASC interacts with pro-caspase-1 and induces auto-cleavage to form a platform, i.e., inflammasome, that stimulates maturation and secretion of IL-1β and IL-18, leads to pyroptosis, a type of programmed cell death [45]. The importance of NLRP3 inflammasome has been implied in many diseases of the heart [57], lung [58,59], liver [54], and kidney [50,60–62]. EGCG has been reported to inhibit NLRP3 inflammasome, and subsequently IL-1β expression in human umbilical vein endothelial cells exposed to palmitate [63], and lupus nephritis [22]. Heme, the substrate of HO-1, can also activate the NLRP3 inflammasome [31,45]. Therefore we studied the effect of EGCG on the NLRP3 inflammasome, which promotes the maturation of IL-1β. EGCG down-regulated NLRP3 expression, which was blocked by ZnPP, indicating that HO-1 links EGCG with NLRP3, and thereby, decreasing the expression of IL-1β.

**Fig 5:**
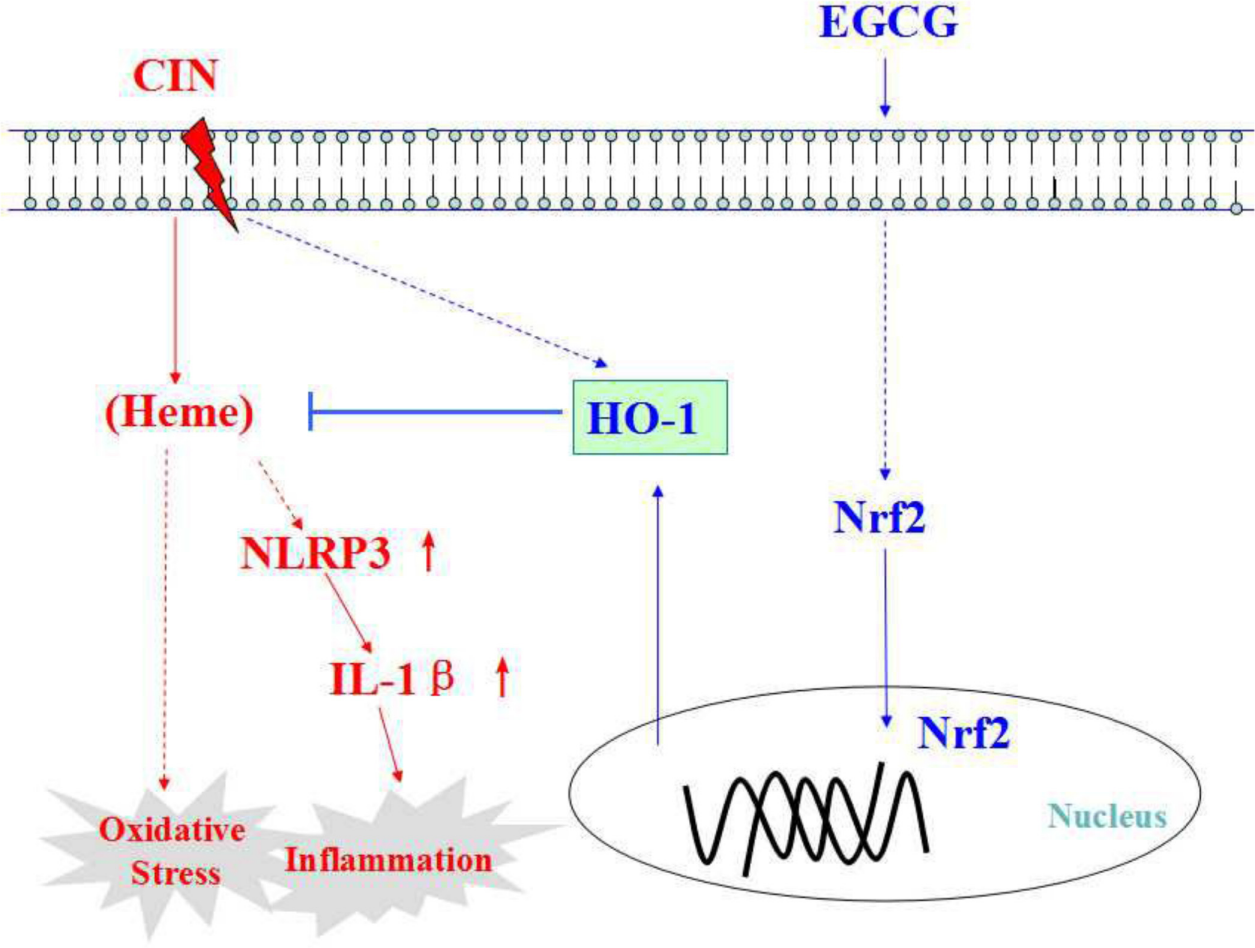
Schematic diagram of possible mechanism of EGCG in protecting against CIN. HO-1 expression is markedly enhanced by EGCG via activation of the Nrf2/HO-1 pathway. HO-1 is essential to the protective effect of EGCG on CIN in anti-oxidation and anti-inflammation. Dash-line arrow stands for unclear or multiple-step actions. Straight-line arrow stands for direct actions.

In conclusion, the peri-operative intravenous administration of EGCG could protect against CIN. HO-1 is key to the EGCG-mediated protection because blockade of HO-1 abolished the down-regulation of reactive oxygen species production and inflammation caused by EGCG (Fig 5).

## Supporting Information

S1 Fig Demonstration of the histological injury site of the CIN model. Four portions of the kidney section stained by H&E: cortex, conjunction of cortex and medulla, outer medulla and inner medulla, both in vehicle and CM groups were shown. The major damage was located in the outer medulla (mTALs, medullary thick ascending limb). The cortical convoluted segment and pars recta segment (S3) of the proximal tubule with apparently larger cell morphology and longitudinal arrangement in the conjunction were almost intact.
(TIF)

S2 Fig Histological distribution of HO-1 induced by EGCG treatment of CIN. Immunofluorescence microscopy of HO-1in kidney demonstrated that HO-1 was mainly expressed in tubules of medulla in the vehicle group; after CM-induced AKI, HO-1 was significantly increased in tubules both in the cortex and medulla; EGCG treatment further profoundly increased the expression of HO-1 in those tubules both in the cortex and medulla. The glomeruli were consisitently spared in all groups.
(TIF)

S3 Fig Role of HO-1 in renal protective effect of EGCG in CIN attested by SnPP. EGCG (10mg/kg body wt) was intravenously infused 15 min before the establishment of CIN. The HO-1 inhibitor SnPP (10mg/kg body wt) was injected intraperitoneally 2h before EGCG pretreatment. The rats were sacrificed at 24h after the establishment of CIN. Serum Cr (A) and BUN (B) were measured. (n = 5, * P<0.05 vs. vehicle; & P<0.05 vs. EGCG).
(TIF)
