# Supplementary figures and images for: Targeting HO-1 by Epigallocatechin-3-Gallate Reduces Contrast-Induced Renal Injury via Anti-Oxidative Stress and Anti-Inflammation Pathways
Source: PLoS One. 2016 Feb 11;11(2):e0149032. doi: 10.1371/journal.pone.0149032 (PMC4750900; doi:10.1371/journal.pone.0149032)

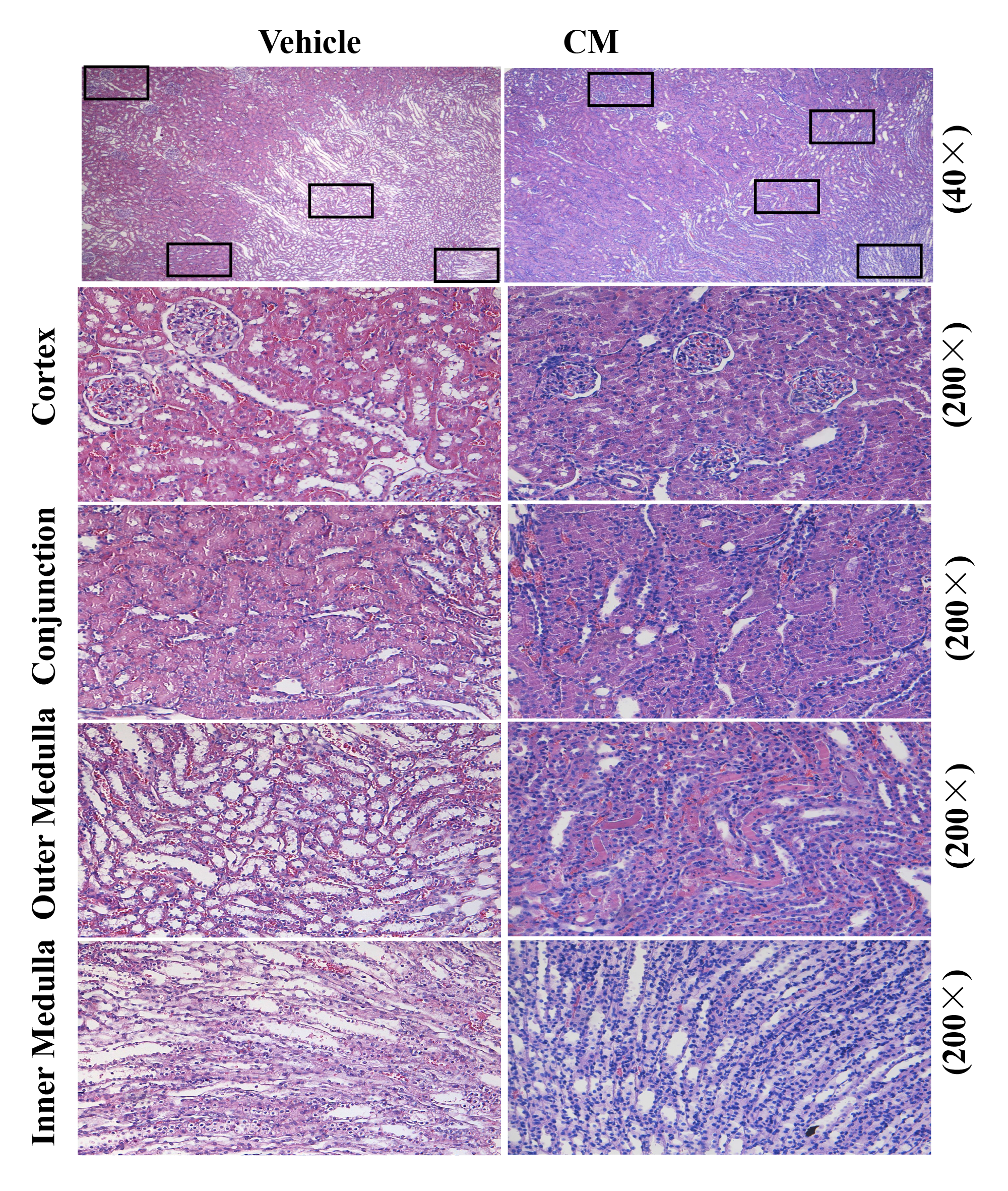

Supplement: S1 Fig — Four portions of the kidney section stained by H&E: cortex, conjunction of cortex and medulla, outer medulla and inner medulla, both in vehicle and CM groups were shown. The major damage was located in the outer medulla (mTALs, medullary thick ascending limb). The cortical convoluted segment and pars recta segment (S3) of the proximal tubule with apparently larger cell morphology and longitudinal arrangement in the conjunction were almost intact. (TIF) [file pone.0149032.s001.tif]

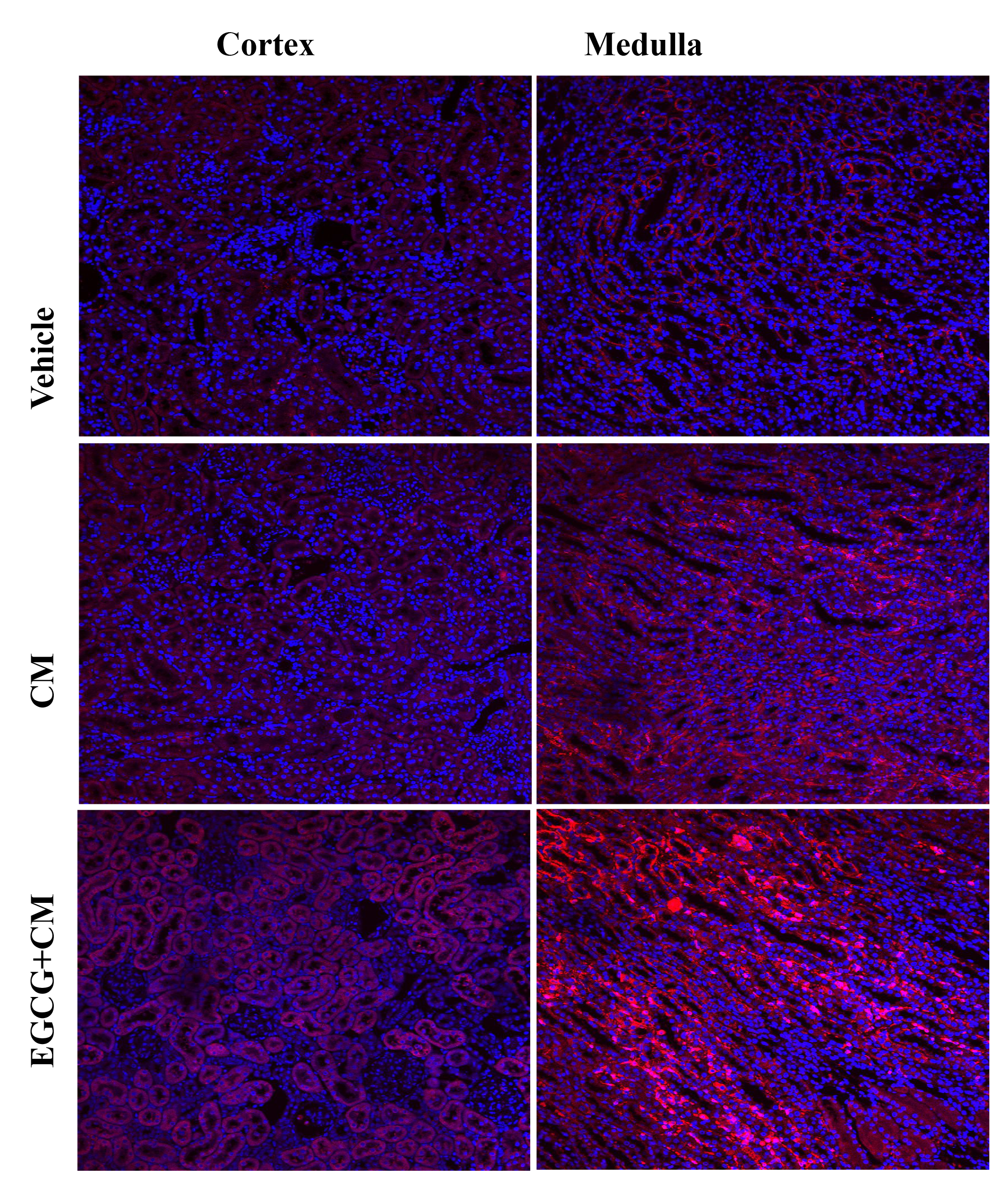

Supplement: S2 Fig — Immunofluorescence microscopy of HO-1in kidney demonstrated that HO-1 was mainly expressed in tubules of medulla in the vehicle group; after CM-induced AKI, HO-1 was significantly increased in tubules both in the cortex and medulla; EGCG treatment further profoundly increased the expression of HO-1 in those tubules both in the cortex and medulla. The glomeruli were consisitently spared in all groups. (TIF) [file pone.0149032.s002.tif]

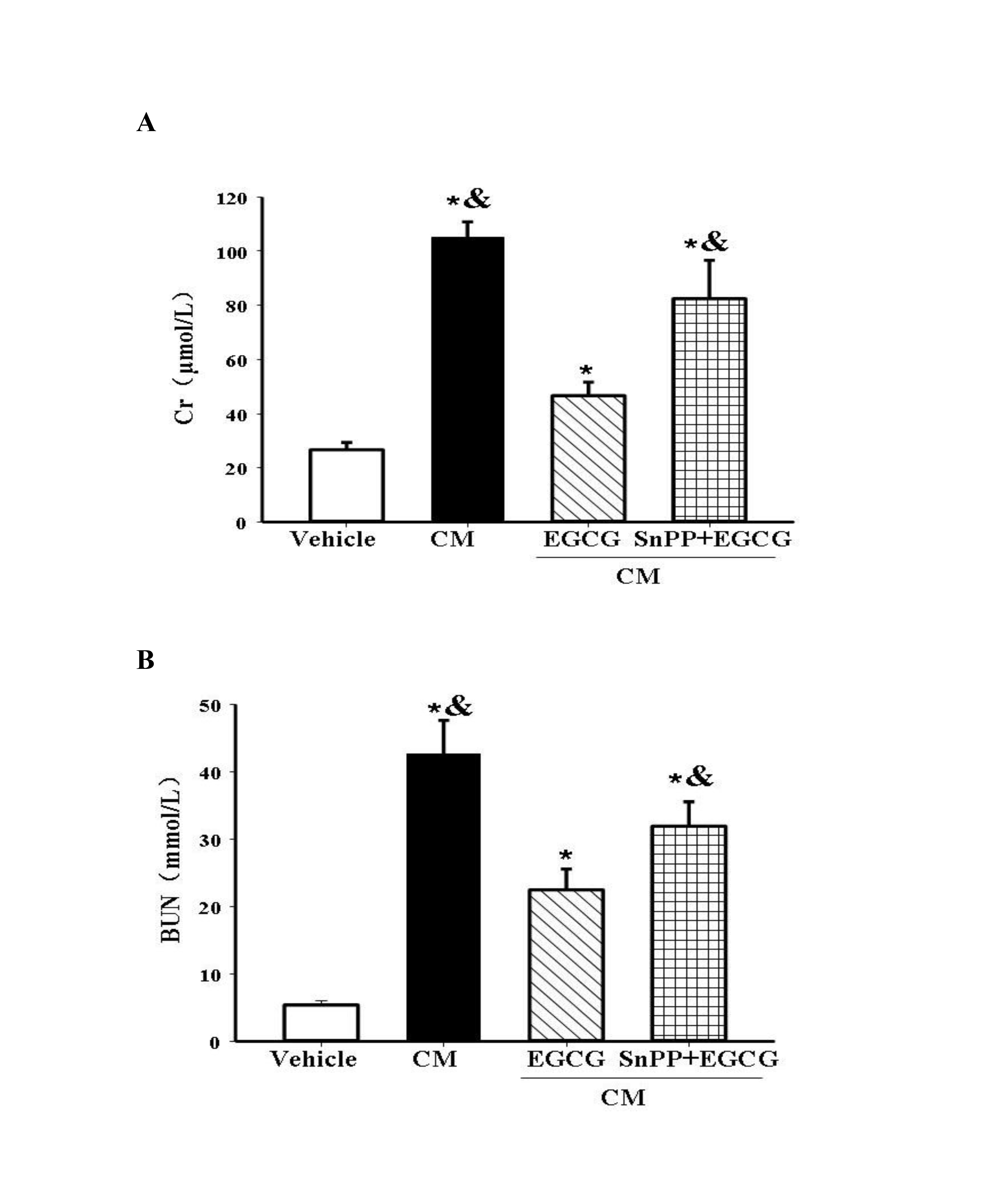

Supplement: S3 Fig — EGCG (10mg/kg body wt) was intravenously infused 15 min before the establishment of CIN. The HO-1 inhibitor SnPP (10mg/kg body wt) was injected intraperitoneally 2h before EGCG pretreatment. The rats were sacrificed at 24h after the establishment of CIN. Serum Cr (A) and BUN (B) were measured. (n = 5, * P<0.05 vs. vehicle; & P<0.05 vs. EGCG). (TIF) [file pone.0149032.s003.tif]
